# Dkk4 and Eda Regulate Distinctive Developmental Mechanisms for Subtypes of Mouse Hair

**DOI:** 10.1371/journal.pone.0010009

**Published:** 2010-04-01

**Authors:** Chang-Yi Cui, Makoto Kunisada, Yulan Piao, Victoria Childress, Minoru S. H. Ko, David Schlessinger

**Affiliations:** Laboratory of Genetics, National Institute on Aging, National Institutes of Health, Baltimore, Maryland, United States of America; Stanford University School of Medicine, United States of America

## Abstract

The mouse hair coat comprises protective “primary” and thermo-regulatory “secondary” hairs. Primary hair formation is ectodysplasin (Eda) dependent, but it has been puzzling that Tabby (*Eda* ^-/*y*^) mice still make secondary hair. We report that Dickkopf 4 (Dkk4), a Wnt antagonist, affects an auxiliary pathway for Eda-independent development of secondary hair. A *Dkk4* transgene in wild-type mice had no effect on primary hair, but secondary hairs were severely malformed. Dkk4 action on secondary hair was further demonstrated when the transgene was introduced into Tabby mice: the usual secondary follicle induction was completely blocked. The Dkk4-regulated secondary hair pathway, like the Eda-dependent primary hair pathway, is further mediated by selective activation of Shh. The results thus reveal two complex molecular pathways that distinctly regulate subtype-based morphogenesis of hair follicles, and provide a resolution for the longstanding puzzle of hair formation in Tabby mice lacking Eda.

## Introduction

Skin appendage formation is regulated by reciprocal signaling between mesenchyme and ectoderm, involving common morphogens such as Wnt, Shh and BMP [1]. Relatively early in evolution, a pathway based on *EDA*, a TNF superfamily member, was interposed downstream of inductive Wnt signaling [2]. The ligand ectodysplasin, in conjunction with receptor EDAR and receptor adaptor EDARADD, activates NF-kB mediated transcription for skin appendage development [3], [4], [5]. In fish, for example, all scale formation depends on *eda*; and in mammals, sweat gland development similarly shows a complete dependence on the *EDA* pathway [6], [7].

However, there has been a puzzling discrepancy for a subgroup of hair follicles in mice and other mammals. In mice, “primary” guard hairs, constituting less than 5% of mouse hair on the back skin, overlay and protect the 95% of “secondary” hairs. Secondary hair, including awl, auchen and zigzag subtypes, have a pivotal physiological role as a thermal insulator, compensating for the lack of sweat glands on the mouse body. Primary and secondary hair follicle formation share some features but also diverge, especially in their degree of dependence on Eda. In *Eda* mutant Tabby mice, no primary hair follicles form, but secondary follicles initiate normally, though they result in straight, thin, short hairs [8], [9].

Mice indistinguishable from Tabby are also produced when other genes in the initial *Eda* receptor/adaptor complex (*Edar* or *Edaradd*) are mutated; and NF-kB knockdown mice display a similar phenotype [4], [5], [10]. Furthermore, when an *Eda-A1* transgene or recombinant ectodysplasin was put into Tabby mice, it fully restored primary hair and sweat glands, and partially restored the form of secondary hair without changing follicle numbers [11], [12].

Consistent with the presence or absence of hair subtypes, Shh pathway genes, which are downstream of Eda/NF-kB [7], [9], [13], were undetectable during the failed primary hair follicle induction stage in Tabby skin [4], but were somehow still activated in the absence of *Eda* during the later formation of secondary hair follicle germs [14], [15]. Therefore, a search for an alternative regulatory loop that activates Shh and initiates secondary hair follicles seemed logical.

In this regard, the Wnt pathway is required to set up the initiation of all types of hair follicles [16], [17], [18], and it is intriguing that several independent studies pointed to a soluble antagonist of Wnt signaling, Dickkopf 4 (Dkk4), that was highly expressed in primary hair follicle germs, but sharply declined in secondary hair follicle germs and growing hair follicles [13], [19], [20]. We thus inferred that Dkk4 may affect hair follicle subtype determination, likely through Wnt signaling, during development. To address the role of Dkk4 in hair follicle development, we generated skin-specific Dkk4 transgenic mice in wild-type and Tabby backgrounds. Unlike primary hair follicle development that solely depends on Eda, we show that secondary hair follicle development is mainly regulated by a Dkk4-regulated pathway; both pathways converge to mediate hair production through the Shh pathway. The results thus reveal distinctive molecular pathways that differentially regulate development of hair follicle subtypes.

## Results

### Primary hairs were normal, but secondary hairs were severely malformed in Dkk4 transgenic mice in wild-type background

**Figure 1:**
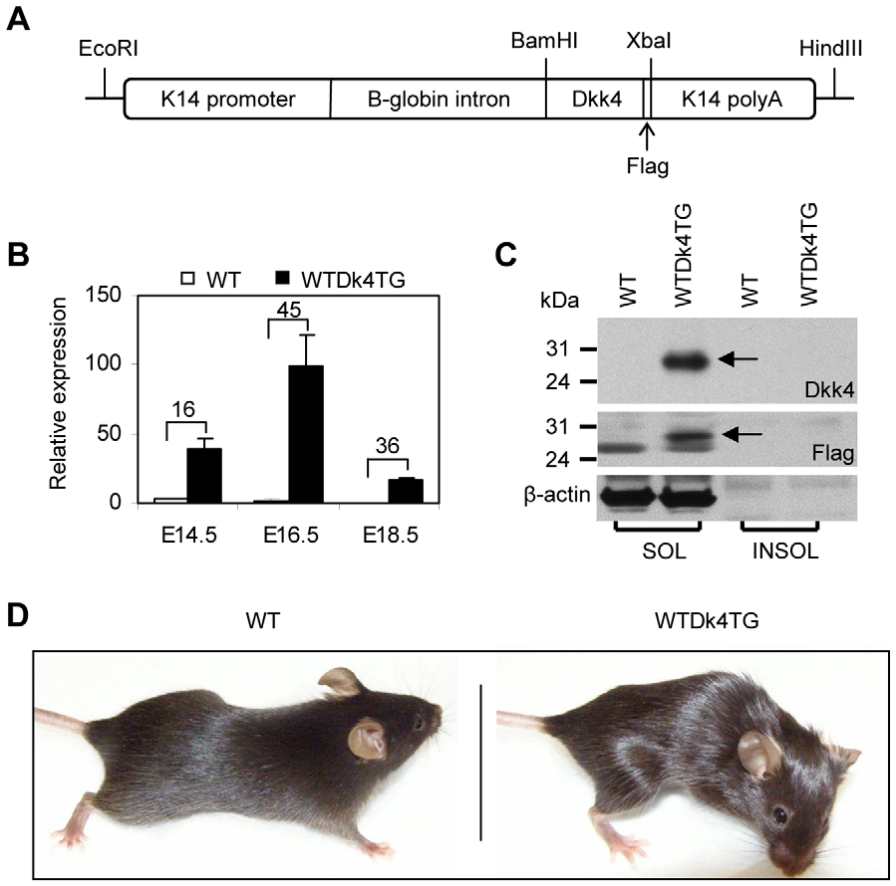
The WTDk4TG mice have a rough hair coat. A, *Dkk4* transgene structure. Full-length mouse *Dkk4* cDNA with a Flag sequence in the 3′ end was inserted into a K14 vector using BamHI and XbaI sites. The linearized EcoRI/HindIII transgene fragment was used for microinjection. B, Transgene expression was sharply up-regulated from E14.5. C, Increased Dkk4 protein production was detected in the soluble fraction (SOL) of WTDk4TG skin at E16.5 in Western blotting analysis with antibodies against Dkk4 and Flag (arrows). D, WTDk4TG mice at 2 months of age. The hair coat in transgenic mice is rough.

To assess the role of Dkk4, we generated a transgenic strain with skin-specific *Dkk4* expression under K14 promoter control (WTDk4TG) (Fig. 1A). Sharply elevated *Dkk4* expression in the back skin of transgenic mice from E14.5 was detectable by Q-PCR assays (Fig. 1B), and Western blotting with anti-Dkk4 and anti-Flag antibodies confirmed the increased expression of Dkk4 protein in the soluble fraction of E16.5 transgenic skin extracts (arrows in Fig. 1C). The transgenic mice were easily distinguished from wild-type littermates by their rough hair coat and abnormal eyes in the adult stage (Fig. 1D).

**Figure 2:**
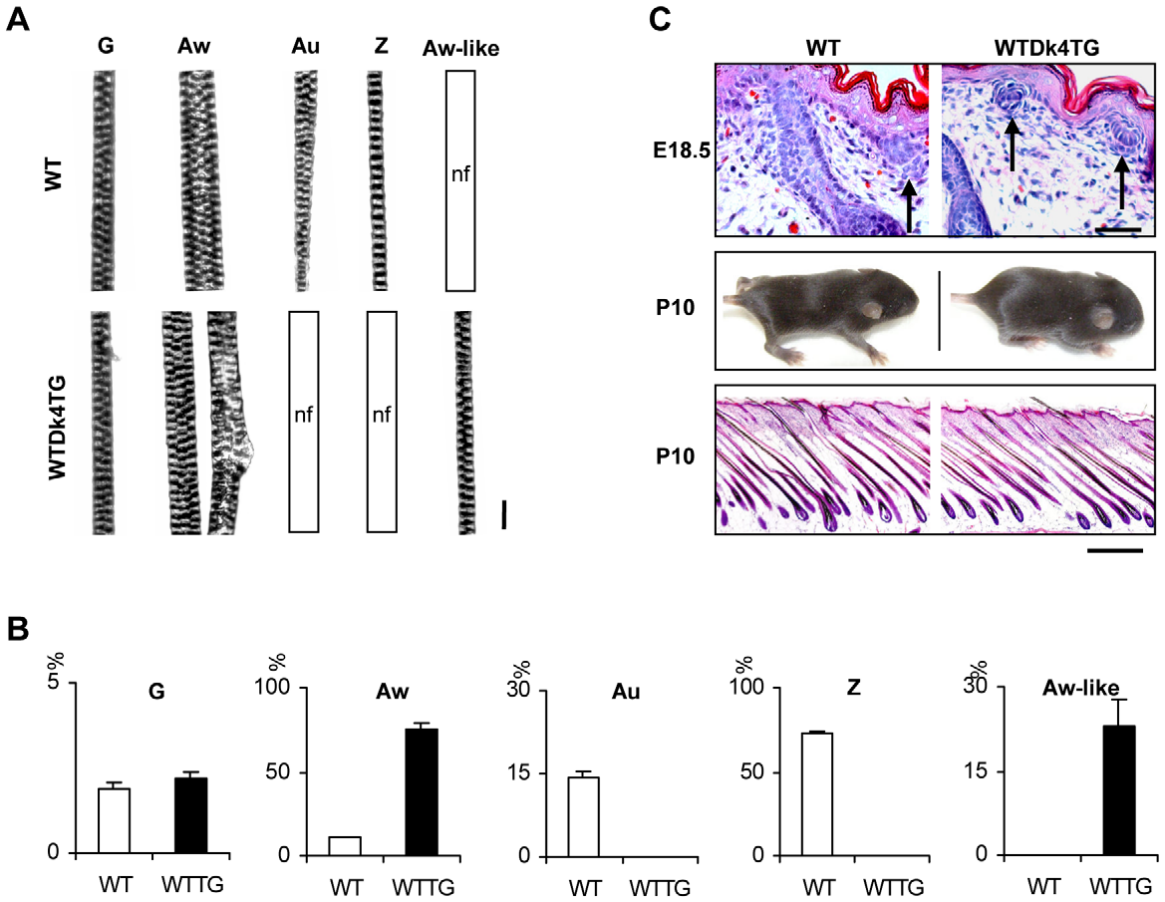
Secondary, but not primary, hairs are severely malformed in WTDk4TG mice. A, Morphology of each hair subtype is shown. In WTDk4TG mice, primary guard hair (G) was indistinguishable from that of WT controls, but awl hair (Aw) was slightly thinner or structurally aberrant, and auchen (Au) and zigzag (Z) hairs were absent. A thin awl-like abnormal hair (Aw-like) was formed in WTDk4TG mice. nf: not found. Scale bar, 200 µm. B, Quantitative analyses of hair subtypes. Primary hair numbers in WTDk4TG mice were comparable to WT controls. Awl hair numbers were significantly increased, auchen and zigzag hairs were absent, and awl-like abnormal hairs were newly formed in Dkk4 transgenic mice. C, Auchen/zigzag hair follicle germs were formed at E18.5 both in WT and WTDk4TG mice (arrows in upper panels). The density of the hair coat in WTDk4TG mice was indistinguishable from that of WT littermates at P10 (middle panels). Comparable follicle numbers between WTDk4TG and WT mice at P10 were observed in histological analyses (lower panels). Scale bars, upper panels, 400 µm; lower panels, 1000 µm.

Notably, the numbers, structure and size of primary hairs (G) in WTDk4TG mice were indistinguishable from wild-type (WT) littermates (Fig. 2A). In contrast, secondary hairs were severely malformed. Awl hairs (Aw) were slightly thinner or structurally aberrant (Fig. 2A). Further, their numbers were significantly increased (Fig. 2B). Also, as in Tabby (Ta) mice, bent zigzag (Z) and auchen (Au) hair types were completely absent (Fig. 2A, B). Instead, awl-like straight short thin secondary hairs (Aw-like) were formed in transgenic mice, accounting for ∼23% of the total hair follicles (Fig. 2A, B).

Histological studies showed that zigzag/auchen follicle germs were induced in transgenic mice at E18.5, as in WT (Fig. 2C, arrows in upper panels). Also, total follicle numbers in transgenic mice were comparable to WT littermates analyzed at postnatal day 10 (P10), both grossly and microscopically (Fig. 2C, middle and lower panels). Thus, normal numbers of hair follicles were initiated, but they produced abnormal secondary hair.

**Figure 3:**
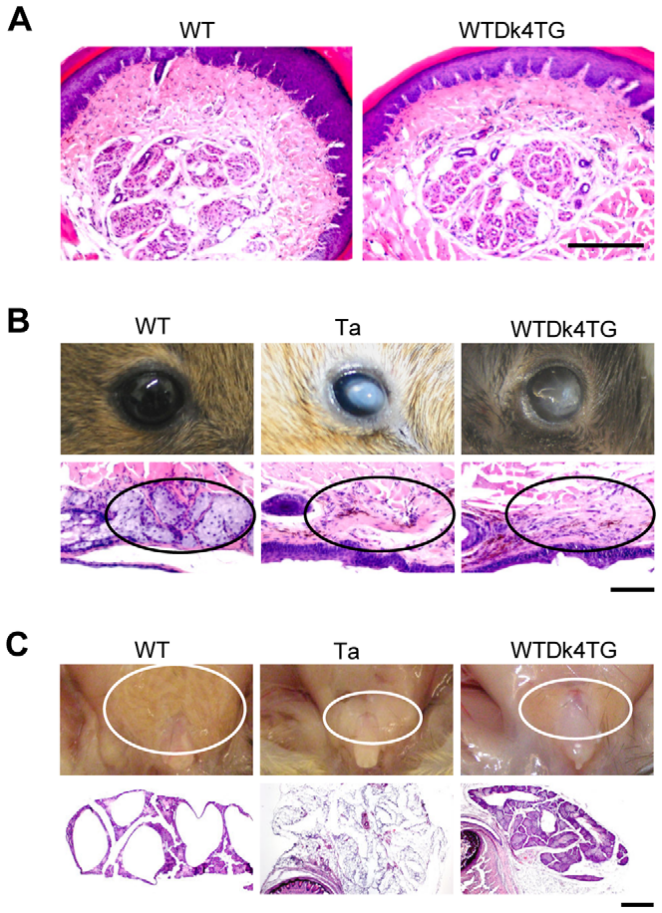
Skin exocrine gland formation was selectively regulated by Dkk4. A, H&E staining shows normally formed sweat glands in adult stage *Dkk4* transgenic footpads, which are indistinguishable from wild-type controls. Scale bar, 200 µm. B, *Dkk4* transgenic mice develop cataracts detectable at around 6 months old (upper panels). Like Tabby, *Dkk4* transgenic mice lack meibomian glands (lower panels). Scale bar, 100 µm. C, Preputial glands in *Dkk4* transgenic mice were about 1/3 of WT control in size (upper panels). Histological analyses showed matured glandular tissue in wild-type mice, absence of glandular tissue in Tabby and smaller, less developed glandular tissue in *Dkk4* transgenic mice (lower panels). Scale bar, 500 µm.

We further found that skin exocrine gland formation was also selectively regulated by Dkk4. Sweat glands were normally formed in WTDk4TG mice, suggesting their development, like primary guard hair, is Dkk4-independent (Fig. 3A). However, like Ta mice, the transgenic mice lacked meibomian glands associated with their eyelids and developed visible cataracts at around 6 months of age, suggesting that meibomian gland development is Dkk4-responsive (Fig. 3B). Preputial gland formation was also affected by Dkk4 levels. The glands were only about 1/3 WT size in the transgenic mice, and histological studies revealed only primitive gland tissue (Fig. 3C).

We further focused on the selective action of Dkk4 in hair follicle development. To identify genes involved in the formation of the aberrant secondary hairs, we carried out expression profiling of WT and WTDk4TG skin at various developmental stages. A number of terminal differentiation markers of hair follicles, including hair follicle-specific keratins, were significantly down-regulated in transgenic skin at late developmental stages, E18.5 and P1, and hair keratin-associated proteins were also down-regulated at P1 (Fig. S1). There was a progressive later increase of significantly affected genes from the small number affected at E14.5, but the additional genes affected, for example, at E16.5, did not include genes known to be involved in hair follicle development or epidermal differentiation. They may speculatively rather reflect aberrant dermal-fatty layer formation seen in TaDkk4TG mice (see below).

### A Dkk4 transgene completely blocked secondary hair follicle induction in Tabby mice

**Figure 4:**
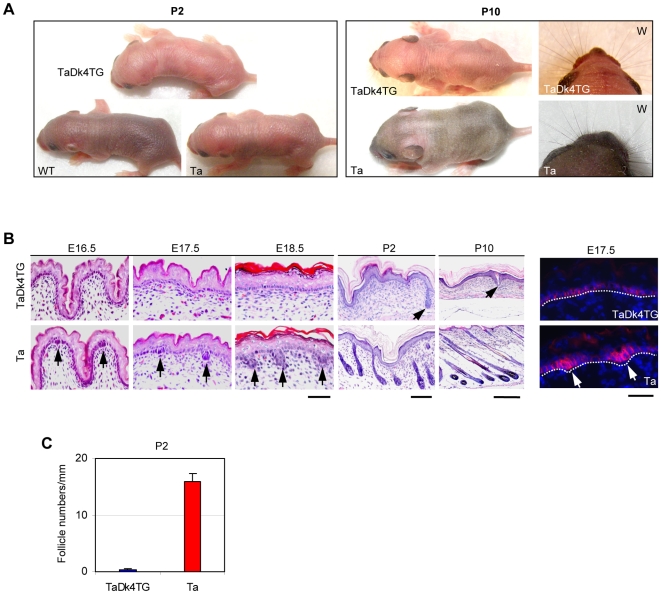
A *Dkk4* transgene completely blocked hair follicle induction in Tabby mice. A, Growing hair made WT pups black and Ta pups grey, but TaDk4TG pups were pink with thin and translucent skin at P2. At P10, TaDk4TG pups were complete hairless, but Ta pups showed a dense yellowish hair coat. Notably, TaDk4TG pups developed normal whiskers (W in right corner) as did Ta. B, Histological progression of hair follicle development in Ta and TaDk4TG mice. Hair follicle germs were discernible at E16.5 and grew down thereafter (arrows in lower panels), stage 4 to 5 hair follicles were seen at P2, and stage 7 to 8 follicles were clear at P10 in Ta mice (lower right panel). Hair follicle induction was not detected in TaDk4TG mice in the embryonic stages, but a late-forming hair follicle was occasionally found at P2, and an epidermal invagination was seen at P10 (arrows in P2 and P10). TaDk4TG skin lacked a fatty layer at P10. Immunofluorescent staining of P-cadherin confirmed hair germ formation in Ta at E17.5 (arrows in right panels), but not in TaDk4TG embryos. Scale bars for embryos, 400 µm; for P2, 1000 µm; for P10, 200 µm; for P-cadherin, 50 µm. C, The retarded hair follicles formed in TaDk4TG mice numbered less than 2% of the hair follicles in Ta littermates.

Results in WTDk4TG mice thus were consistent with the hypothesis that Dkk4 selectively affects secondary hair follicle development. To focus more precisely on Dkk4 function in secondary hair follicles, we introduced the *Dkk4* transgene into Ta mice, the “pure” model for secondary hair follicle development. The resulting *Dkk4* transgenic Tabby (TaDk4TG) pups usually die before day 2 after birth (P2), though a few mice survive up to P10. Grossly, Ta back skin appeared grayish because of hair growth at P2, but TaDk4TG skin remained pink, thin and translucent, and the animals were thus easily distinguishable from Ta or black WT littermates (Fig. 4A). At P10, WT mice were covered by black hair as shown in Fig. 2C, Ta mice formed a dense uniformly short yellow hair coat, but TaDk4TG mice were completely hairless (Fig. 4A). Notably, in contrast to body hair, whiskers were normally formed in TaDk4TG mice by P10 (Fig. 4A).

Histological studies showed that early stage hair follicle germs were discernible at E16.5; late stage hair follicle germs were visible at E17.5; and stage 2 hair follicles were clear at E18.5 in Ta mice (Fig. 4B). In sharp contrast, no hair follicle germs were observed in TaDk4TG mice at any embryonic stages analyzed. Absence of hair follicle induction in TaDk4TG skin at E17.5 was confirmed by immunofluorescent staining for P-cadherin, an early stage hair follicle marker (Fig. 4B, right panels). By P2 in Ta mice, hair follicles entered stage 4–5, characterized by formation of the dermal papillae (Fig. 4B). However, only an occasional hair follicle at about early stage 2 was observed in TaDk4TG mice (Fig. 4B). The late hair follicles seen in TaDk4TG mice at P2 amounted to less than 2% of those in Ta (Fig. 4C). By P10, hair follicles entered stage 7 to 8 producing hair shafts in Ta, but no follicles were found in TaDk4TG mice (Fig. 4B, P10). We found very occasional epidermal invaginations, probably derived from the few delayed follicles seen at P2. Notably, skin fatty layer was absent in TaDk4TG skin (Fig. 4B, P10). Based on these results, we conclude that Dkk4 demonstrably regulates early stage induction as well as later differentiation of secondary hair follicles.

### A Dkk4 transgene did not affect EDA pathway genes, and was unable to rescue Ta phenotypes

**Figure 5:**
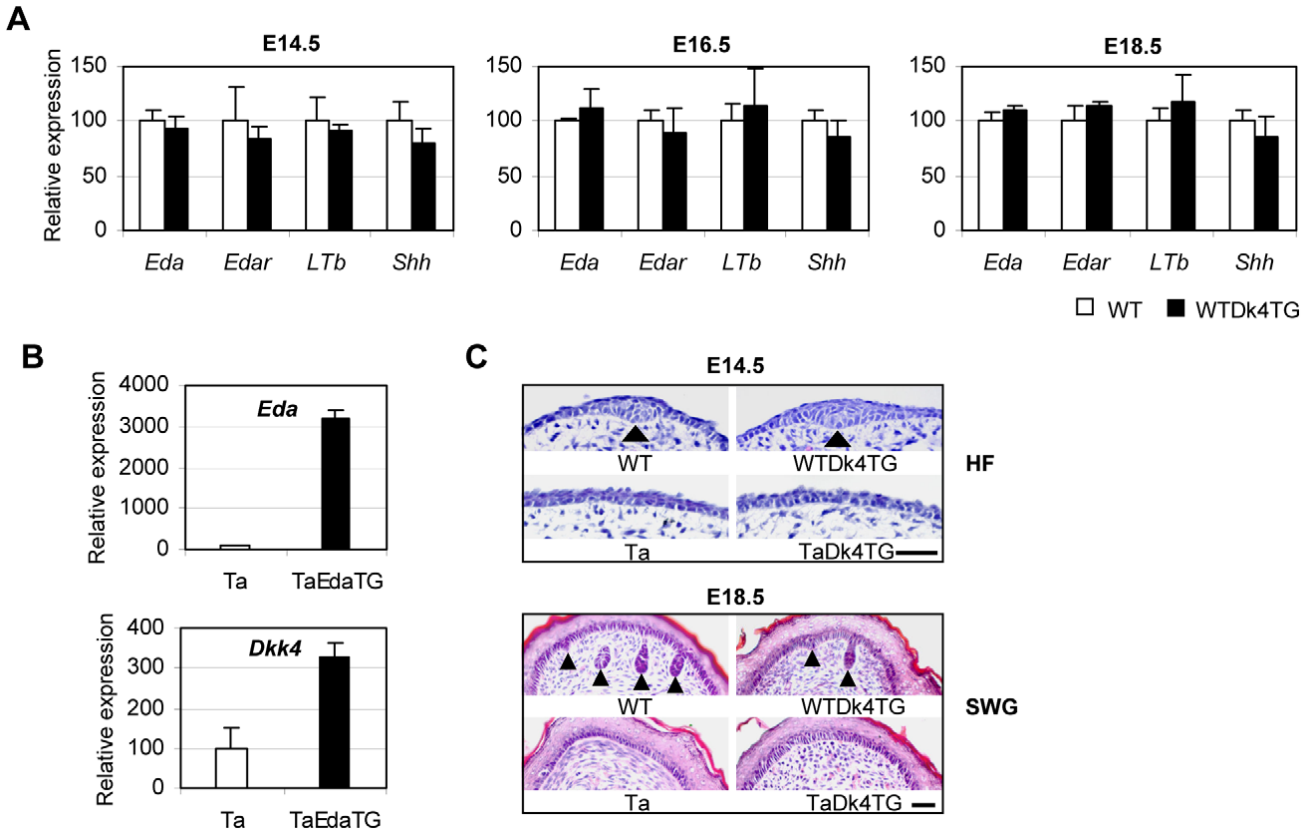
*EDA* pathway genes were not affected in *Dkk4* transgenic mice, and the *Dkk4* transgene did not rescue Ta phenotypes. A, Q-PCR assays showed that expression levels of *Eda, Edar, LTb* and *Shh* were not changed in WTDk4TG skin at E14.5, 16.5 and 18.5. B, Expression levels of *Eda* (upper panel) and *Dkk4* (lower panel) were upregulated in *Eda-A1* transgenic Tabby mice (TaEdaTG) at E16.5. C, Primary hair germs were normally formed in WT and WTDk4TG mice, but not in Ta or TaDk4TG mice, at E14.5 (upper panels). Similarly, sweat gland pegs were normally formed in WT and WTDk4TG mice, but not in Ta or TaDk4TG mice at E18.5 (lower panels). Scale bars, 400 µm.

The partially Ta-like phenotypes seen in WTDk4TG mice prompted us to analyze possible regulatory interactions between Dkk4 and Eda. Wnt function has been implicated upstream of *Eda* [2], [14], and a *Dkk1* transgene inhibited expression of the Eda receptor *Edar* in mice [16]. To assess whether *Dkk4* action in transgenic mice was mediated by a Wnt-Eda cascade, we examined expression levels of the *EDA* pathway genes *Eda, Edar, Shh* and *LTb* [21], [22] in WTDk4TG mice (Fig. 5A). However, consistent with microarray results, Q-PCR assays showed no significant expression changes for these genes in transgenic skin at any embryonic stages. Thus, the Ta-like secondary hair phenotypes seen in WTDk4TG mice appear to be essentially Eda-independent (Fig. 5A).

*Dkk4* was previously shown to be down-regulated in Ta embryonic skin [13], and was up-regulated by recombinant ectodysplasin added to organ cultures of Ta skin [23]. To assess further whether Dkk4 is a downstream target of *Eda in vivo*, we collected E16.5 back skin from Ta and *Eda-A1* transgenic Tabby mice (TaEdaTG) [24]. By Q-PCR, we found a 3-fold up-regulation of *Dkk4* expression in TaEdaTG skin (Fig. 5B). Though *Dkk4* up-regulation by *Eda in vivo* was more moderate than *in vitro* [23], the results are consistent with that Eda as a likely upstream regulator of Dkk4.

To see if supplementation of *Dkk4* in Ta mice was able to restore Ta phenotypes, we further analyzed development of the two major target appendages of *Eda,* primary guard hair and sweat gland germs, in TaDk4TG and WTDk4TG embryos. Primary guard hair germs were induced normally in WT and WTDk4TG at E14.5, but not in Ta or TaDk4TG littermates (Fig. 5C). Similarly, sweat gland pegs were evident in WT and WTDk4TG footpads at E18.5, but not in Ta or TaDk4TG littermates (Fig. 5C). We conclude that 1) even though expression levels are sharply elevated from an early stage, a *Dkk4* transgene does not affect induction of guard hair follicles or sweat glands in WT mice–consistent with phenotypic observations in adult stage transgenic mice; and 2) as expected, *Dkk4* supplementation in Ta mice does not rescue guard hair follicles or sweat glands.

Thus, Dkk4 acts neither by a feedback inhibitory effect on Eda, nor by a simple mediation of morphogenetic effects of Eda.

### Shh, but not other morphogens, was absent in TaDk4TG mice during secondary hair follicle induction

**Figure 6:**
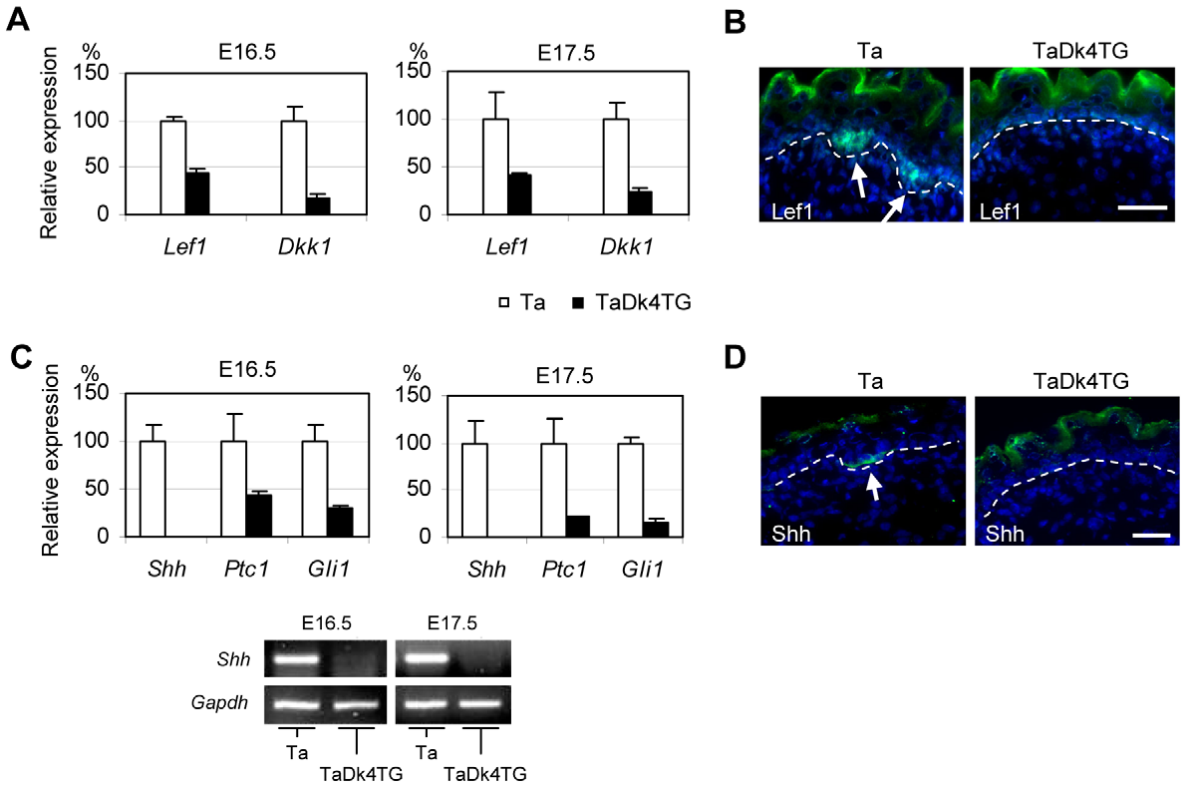
Wnt and Shh pathway genes were significantly downregulated in TaDk4TG skin. A, Q-PCR assays confirmed the significant downregulation of Wnt effector Lef1 and Wnt target Dkk1 in TaDk4TG skin at E16.5 and E17.5. B, Immunofluorescent staining revealed a nuclear localization of Lef1 protein in hair follicle germs in Tabby skin at E17.5 (arrows), but not in TaDk4TG skin. Scale bar, 50 µm. C, Shh was undetectable, and Ptc1 and Gli1 were significantly down-regulated, in TaDk4TG skin at E16.5 and E17.5, as assessed by Q-PCR (upper panels). Lower panels, electrophoresis of Q-PCR products after 40 cycles of amplification confirmed the absence of *Shh* in TaDk4TG. D, Shh protein was localized in the membrane and cytosol of the apical surface of hair follicle germs in Ta skin at E17.5, but was not seen in TaDk4TG. Scale bar, 50 µm.

Although secondary hair formation responds primarily to an Eda-independent initiating mechanism, major downstream effectors are shared. To detect genes involved in Dkk4-responsive secondary hair follicle induction, we did expression profiling of Ta and TaDk4TG skin at E16.5 and E17.5. Full lists of genes affected at E16.5 and expression changes of corresponding genes at E17.5 are shown in Table 1 (Fig. S2 gives a full list of genes affected at E17.5). Among the small numbers of altered genes, the Wnt effector Lef1 and the Wnt target Dkk1 were significantly down-regulated in TaDk4TG mice at both time points (Table 1, Fig. 6A). In immunofluorescent staining, Lef1 was normally expressed in the hair follicle germs in Ta mice at E17.5, but absent in TaDk4TG mice (Fig. 6B). Based on these results, the Flag-tagged Dkk4 transgenic protein appears to function by suppressing a canonical Wnt signaling. To look for any affected Wnt pathway genes expressed in skin [25], [26], we further carried out Q-PCR assays with 10 Wnt ligand genes (Wnt3, 3a, 4, 5a, 6, 7a, 7b, 10a, 10b and11), 10 Frizzled receptor genes (Fzd1-10), and 4 co-receptor genes including Lrp5/6 and Kremen1/2. Consistent with Dkk4 action downstream of the Wnt complex, these genes, apart from a marginal up-regulation of Wnt3a, showed no detectable changes in TaDk4TG skin at E16.5 (Table S1).

**Table 1:** Affected genes in TaDk4TG skin at E16.5 and E17.5.

Genes	Fold-Differences (Ta/TaDk4TG)	
	E16.5*	E17.5**
*Shh*	27.5	59.8
*Ptch1*	2.4	5.0
*Ptch2*	2.9	4.4
*Gli1*	3.0	4.0
*Lef1*	2.3	2.4
*Dkk1*	4.6	5.3
*Lgr6*	3.8	3.4
*Tmem16e*	2.9	0.9
*Scube1*	1.7	1.7
*Cxcr4*	1.7	2.3
*Tcf7*	1.7	2.4
*Rgs2*	1.6	1.5
*Id3*	1.6	1.2
*Gprasp2*	1.6	1.0
*ND6*	1.5	0.8
*OTTMUSG00000003947*	1.5	1.2
*Rhpn2*	1.5	2.1
*3110082D06Rik*	1.5	1.3
*Dkk4*	0.05	0.05
*Itgbl1*	0.5	0.7
*6430704M03Rik*	0.6	0.8
*Col8a1*	0.6	0.6
*Agrp*	0.6	0.6
*Sphkap*	0.6	0.7
*E030049G20Rik*	0.6	1.0

*The full list of significantly affected genes at E16.5 is shown.

**The full list of affected genes at E17.5 is listed in the Fig. S2.

The only morphogen downstream of Wnt that was appreciably affected was *Shh* (Table 1, Fig. S2). We found that four Shh pathway genes, *Shh, Ptc1, Ptc2* and *Gli1*, were profoundly down-regulated in TaDk4TG mice at both E16.5 and E17.5. In Q-PCR assays, *Shh* expression in TaDk4TG back skin was undetectable, and *Ptc1* and *Gli1* were significantly down-regulated (Fig. 6C). In immunofluorescent staining, Shh was located in the basal surface of hair follicle germs, adjacent to the basement membrane in Ta mice, but not in TaDk4TG skin (Fig. 6D). Thus, in the absence of *Eda*, Dkk4 blockage of secondary hair follicle induction occurs along with suppression of Shh action.

### Dkk4 action is independent of known effectors of secondary hair follicle formation

Thus far, 3 mesenchymally expressed proteins, Sox2 and Sox18, the Sox family transcription factors, and Noggin, a BMP antagonist, have been shown to be involved in secondary hair follicle development [27], [28], [29]. Sox2-/CD133+ cells were shown to specify zigzag hair, the major secondary hair type [27]. Sox18 mutant ragged mice selectively lose auchen and zigzag hairs [28], and awl, auchen, zigzag hairs were missing in Noggin knockout skin [30]. A recent study also showed that Troy, an Edar family receptor, selectively blocked awl hair follicle induction when mutated in Tabby mice [31]. To assess whether Dkk4 action is further mediated by these effectors, we analyzed their expression levels in WT, Ta and TaDk4TG skin at E16.5.

In Q-PCR assays, Sox2 and Sox18 were significantly down-regulated in Ta skin at E16.5, and TaDk4TG skin showed an expression level comparable to Ta for both genes (Fig. S3). In contrast, CD133 expression was unaffected in Ta or TaDk4TG skin (Fig. S3). Noggin and Troy expression in Ta and TaDk4TG skin was also comparable to WT controls (Fig. S3). Collectively, our data suggest that Dkk4 action in TaDk4TG mice is independent of Sox2, Sox18, Noggin and Troy.

## Discussion

**Figure 7:**
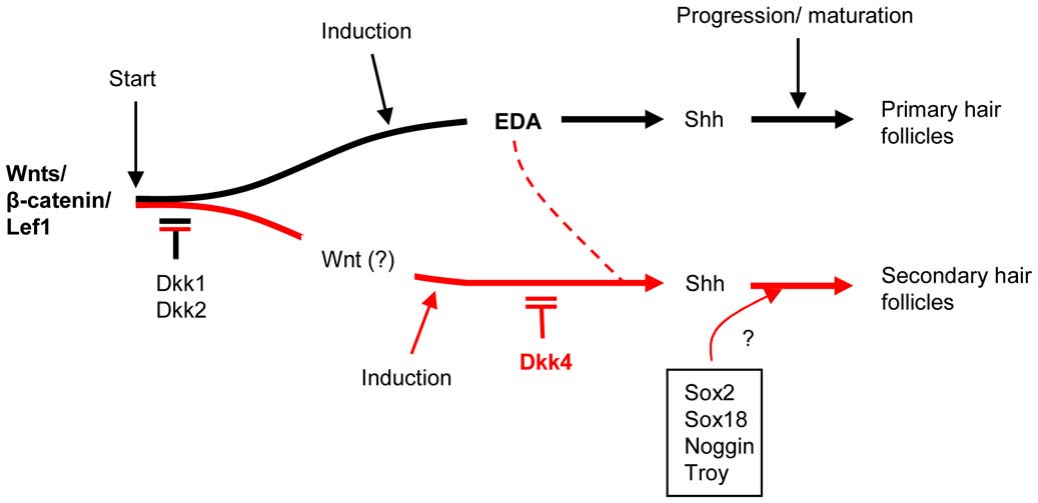
A schematic representation of the hypothesis for differential regulation of hair follicle subtype formation. Wnt/β-catenin signaling is responsible for the development of all subtypes of hair follicles, a process that can be completely blocked by Dkk1 or Dkk2. Primary hair follicle formation is solely dependent on the Wnt-Eda-Shh cascade. A Dkk4-dependent pathway (red lines) regulates secondary hair follicle induction and differentiation, which is further mediated by Shh. Eda plays a modulatory role, as yet undefined in detail, in this process. Sox2, Sox18, Noggin and Troy may also regulate secondary hair follicle development, independent of Dkk4 action.

The study of characteristic hair phenotypes in Ta mice, in which *Eda* is absent, has helped to distinguish similar but distinct molecular mechanisms for the development of different hair subtypes. The canonical Wnt pathway has been demonstrated to be required for all hair follicle initiation, and thus major Wnt inhibitors Dkk1 and Dkk2 block all hair formation [16], [17], [18], [20]. Downstream, a major morphogen cascade, unequivocally dependent on Eda, has been established for primary hair follicles. In contrast, for the more populous secondary hair development, we infer a branch pathway (Fig. 7). A Dkk4-regulated pathway is interposed to activate downstream Shh, and Eda has a modulating function. Here we review the information about Dkk4 action in hair follicle development.

### Selective role of Dkk4 for secondary hair follicle development

Three of the 4 Dkk family members, Dkk1, 2 and 4, inhibit Wnt signaling [32]. Dkk1 and Dkk2 localize to mesenchyme surrounding hair follicle germs in early developmental stages [16], [33]. By contrast, Dkk4 has been found to be expressed only in the epidermal part of skin appendages, and was suggested to regulate hair follicle spacing [19], [20], [23]. Skin-specific *Dkk1* (or *Dkk2*) over-expression inhibited the formation of all subtypes of hair follicles, suggesting that they may affect a universal program early in hair follicle determination [16], [20]. By contrast, *Dkk4* over-expression under the same K14 promoter affected only secondary hair follicle development (Fig. 1, 2). In fact, the expression pattern of endogenous *Dkk4* during normal development correlates inversely with secondary hair follicle formation [13], [19], [20]. A simple interpretation would be that Dkk4 down-regulation at late stages during normal development can enable a Wnt subset(s) to be active and promote secondary hair follicle induction and further development. The secondary hair follicle formation is disrupted if *Dkk4* expression continues from a transgene. Thus, Dkk4 may play a more specialized, delimited role than Dkk1 or Dkk2. Consistent with such a role, current genome databases show that Dkk1 and Dkk2 are highly conserved from fish to human, but Dkk4 is found only in mammals.

As for their mode of action, Dkks do not directly interact with Wnts, but form a complex with Wnt co-receptors Lrp5/6 and Kremen1/2 to inhibit canonical Wnt signaling [32]. Among about 20 Wnt family members, at least 10 are expressed in hair follicles [25]. Individual Wnts were shown to play distinct role for hair or feather development and it was proposed that it may be regulated by multiple factors including secreted Wnt inhibitors [34]. The down-regulation of Wnt effector Lef1 and Wnt target Dkk1 in TaDk4TG mice suggests that Dkk4 most likely affect a subset(s) of canonical Wnt signaling, and further operates through an effect on Shh activation (see below). However, until the putative Wnt subset(s) interacting with Dkk4 is identified, it cannot be excluded that Dkk4 action in transgenic mice may simply reflect different levels of Wnt activities required to generate each hair subtype.

Dkk4 expression was also reported in human esophageal epithelium [35], and was up-regulated in endometrial and colon cancer tissues [36], [37]. In colon cancer cells, Dkk4 was shown to promote cell migration in a Wnt-independent cascade [37], so that an action on hair follicle development through a Wnt-independent pathway cannot be completely excluded at present.

One striking phenotype of WTDk4TG mice was the absence of bends in hair. Because total follicle numbers were unchanged, bent hairs most likely were replaced by straight hairs in WTDk4TG mice. It was recently reported that a Noggin transgene stimulated proliferation of follicle matrix cells, which resulted in replacement of bent hairs by awl-like straight hair [38]. Levels of Igfbp5 and Igf-1 have also been shown to regulate hair bending [39], [40]. However, these candidate regulatory genes showed no significant changes in expression in our microarray profiles (Fig. S1, S3). Thus, further studies are needed to understand the apparent blockage of hair bending in WTDk4TG mice.

The Dkk4 transgene in a wild-type background modulated secondary hair formation to a lesser degree than in Ta mice. The differential effect in WT vs. Ta mice may reflect the interaction of two pathways. Wnt signaling activates *Eda* and *Edar* [2], [41], and Wnt inactivation suppresses the *EDA* pathway in mice, especially during primary guard hair follicle induction [2], [14], [16], [42]. Conversely, the *EDA* pathway was shown to repress Wnt in cell lines [43], and Dkk4 was shown to be an Eda target [13], [23], [41]. This potentially could create a feedback loop between Eda and Wnt [21]. However, Dkk4 is a direct downstream target of Wnt [19], [20], [23], so that Dkk4 is not solely regulated by Eda. Consistent with a more complex interaction, *Dkk4* over-expression did not affect *Eda in vivo*. Our data thus suggest that a Dkk4-repressed pathway plays a major role in the differentiation of secondary hair follicles, but Eda would play a modulatory effect.

### Shh acts downstream of Dkk4 and Eda during hair follicle development

In *Shh* knockout mice, primary hair follicles start to form, but down-growth fails [44]. For secondary hair follicles, the Shh requirement also extends to the stabilization of induction, with knockout mice showing a 60% reduction in the numbers of follicle germs [45]. Recent reports further suggested involvement of Shh in induction of hair follicle germs in addition to be essential for down growth of hair follicles [46], [47].

Shh was the most prominent and most strikingly down-regulated Eda target in Ta hair follicles and sweat glands [7], [13]. In Ta mice it was not expressed during primary guard hair follicle induction stages. However, it was re-expressed in secondary hair follicle germs in Ta mice in late stages [14], [15]; and complete blockage of Shh was seen when a Dkk4 transgene was expressed in Ta. This is consistent with the model that a Dkk4-regulated pathway is responsible for Shh re-expression, which would then enable secondary hair follicle induction in Ta mice. Shh is thus regulated by two distinctive pathways at different developmental stages of hair follicles.

Notably, Shh was the only morphogen detected as down-regulated in TaDk4TG skin in our expression profiling, however, involvement of other morphogens, particularly those with low expression levels at the limit of sensitivity of the approach, cannot be excluded. Indeed, Shh knockout mice showed milder hair follicle phenotypes than TaDk4TG mice, implying the likely function of additional regulators in secondary hair follicle development [44], [45].

Several signaling proteins and transcription factors have been shown to regulate secondary hair follicle development. Secondary hair follicle induction was blocked when Noggin was ablated [30]; and similar to Dkk4, Noggin action was mediated by Lef1 and Shh. However, Noggin showed a broader effect than Dkk4, blocking *Shh* expression in primary follicles and disrupting their differentiation as well [30]. Furthermore, Noggin expression was not affected in Ta or TaDk4TG skin (Fig. S3). Similarly, Troy expression was unchanged in Ta or TaDk4TG mice. Sox2 and Sox18 have also been shown to be involved in secondary hair follicle formation [27], [28], and both were down-regulated in Ta. However, their expression was not further affected in TaDk4TG skin.

Overall, Dkk4 action suggests that Wnt activity is redundant with Eda in secondary hair follicle germs, which provides a resolution for the longstanding puzzle of how secondary hair production can still occur in mammals in the absence of *Eda*. The pathway remains only partially defined, but our data suggest that the Eda-dependent and the Dkk4-responsive pathways regulate subtype-based morphogenesis of hair follicles distinctively and cooperatively through a Shh mediated cascade.

## Materials and Methods

### Ethics Statement

All research was conducted according to relevant national and international guidelines as defined by the Office of Animal Care and Use in the NIH Intramural Program (oacu.od.nih.gov), and all animal study protocols were approved by the NIA Institutional Review Board (Animal Care and Use Committee).

### Generation of skin-specific *Dkk4* transgenic mice in wild-type and Tabby background

The full-length open reading frame of mouse *Dkk4* cDNA (NM_145592.2) was amplified from pCMV-SPORT6-Dkk4 plasmids (Invitrogen) by PCR with a primer set containing a Flag sequence in the reverse primer. Forward: TCTTTTTGGATCCGCCACCATGGTACTGGTGACCTTGCTT. Reverse: GTTTTTTCTAGAGCTACTTGTCATCGTCGTCCTTGTAATCTATTCTTTGGCATACTCTTAGCCTTGA. The transgene was subcloned into a K14 vector using the BamHI and XbaI sites (Fig. 1A). A linear 3.9kB fraction of the K14 promoter/beta-globin Intron/Dkk4 transgene/K14 polyA was cut out by EcoRI and HindIII, purified, and microinjected into pronuclei of one-cell C57BL/6J mouse embryos (Fig. 1A). Microinjected embryos were implanted into pseudo-pregnant female mice. Genotyping was done by PCR with primers spanning Intron 2. Forward: CTCGCTGTGTGCATCA GACA. Reverse: TACTGCTTTGTGATTTCTTCGTA. Potential founders were mated to C57BL/6J mice to identify those passing the transgene. The transgene-positive male progeny (WTDk4TG) were then mated with heterozygous Tabby females (C57BL/6J-Aw-j-Ta6j strain, Jackson Laboratory) to generate Dkk4 transgenic Tabby male mice (TaDk4TG).

### Timed mating, gene expression profiling and Q-PCR assays

Timed mating was set up for K14-Dkk4TG x C57BL/6J, K14-Dkk4TG x Ta, and Eda-A1TG x Ta to get embryos at E14.5, E16.5, E17.5, and E18.5, and newborn mice at P1, P2 and P10 for each strains. The morning after mating was designated as E0.5. Back skin samples and livers were taken, frozen on dry ice, and stored at −80°C until use. Sex and Ta mutation were determined by PCR-based genotyping [13].

Two sets of microarray experiments were carried out: comparison of WT and WTDk4TG at E14.5, E16.5, E18.5 and P1; and comparison of Ta and TaDk4TG at E16.5 and E17.5. Three skin samples from 3 embryos for each genotype at each time point were used for biological replicates. RNA was isolated by Trizol (Invitrogen), precipitated by LiCl, and cyanine-3-labeled cRNAs were generated and hybridized to the NIA Mouse 44K Microarray v3.0 manufactured by Agilent Technologies. Triplicate data were analyzed by ANOVA [7]. Genes with FDR<0.05, fold difference >1.5 and mean log intensity >2.0 were considered to be significant. All data are MIAME compliant and raw data has been deposited in GEO (GSE19309 for the comparison of WT and WTDk4TG; GSE19312 for the comparison of Ta and TaDk4TG).

One-step real-time PCR (Q-PCR) with Taqman probe/primer sets was performed to confirm and extend microarray results (Applied Biosystems). Analyzed genes by Q-PCR include *Eda, Edar, Ltb, Shh, Ptch1, Gli1, Wnt3, 3a, 4, 5a, 6, 7a, 7b, 10a, 10b, 11, Fzd1-10, Lrp5, 6, Kremen1, 2, Lef1, Dkk1, Dkk4, Noggin, Sox2, Sox18,* and *Troy*. Total RNAs from the back skin of E16.5 or E18.5 WT embryos were used to generate a standard curve. Each of the two sets of RNAs for each genotype was assayed in triplicate by Q-PCR. Reactions were normalized to GAPDH.

### Histology, immunohistochemistry and Western blotting

Histology of hair follicles, sweat glands, meibomian glands and preputial glands was analyzed by H&E staining with paraffin sections. Hair subtypes were analyzed with more than 400 hairs for each mouse, and their morphology was scored under a dissection microscope.

For immunofluorescent staining, frozen skin sections (8 µm) were fixed in 100% acetone at −20°C for 10 min, incubated with primary antibodies at 4°C overnight, followed by AlexaFluor secondary antibodies (Invitrogen), and were analyzed under a DeltaVision microscope. Anti-P-cadherin (Invitrogen, 1∶100), anti-Lef1 (Cell Signaling, 1∶100) and anti-Shh (Santa Cruz, N-19, 1∶50) were used as primary antibodies.

For Western blotting, proteins were isolated from E16.5 back skin of WT and WTDk4TG embryos by homogenization in RIPA buffer (Sigma) (the soluble fraction). The pellets were then subjected to RIPA+1%SDS and sonication (the insoluble fraction). Proteins were fractionated in 10% SDS/polyacrylamide gel electrophoresis and then transferred to a nitrocellulose membrane. Anti-Dkk4 antibody (R&D Systems, 1∶500) and anti-Flag M2 antibody (Sigma, diluted to 10 µg/ml) were used as primary antibodies and the reactive bands were detected via an ECL kit (Amersham Life Sciences).

## Supporting Information

Figure S1 The full list of differentially expressed genes between WT and WTDk4TG skin
(0.05 MB PDF)

Figure S2 The full list of differentially expressed genes between Ta and TaDk4TG skin
(0.05 MB PDF)

Figure S3 Expression levels of Sox2, Sox18, CD133, Noggin and Troy in Ta and TaDk4TG skin at E16.5.
(0.03 MB PDF)

Table S1 Expression levels of Wnt pathway genes in Ta and TaDk4TG skin at E16.5
(0.04 MB DOC)
