# Supplementary material for: Dkk4 and Eda Regulate Distinctive Developmental Mechanisms for Subtypes of Mouse Hair
Source: PLoS One. 2010 Apr 1;5(4):e10009. doi: 10.1371/journal.pone.0010009 (PMC2850388; doi:10.1371/journal.pone.0010009)
Supplement: Figure S1 — The full list of differentially expressed genes between WT and WTDk4TG skin (0.05 MB PDF) [file pone.0010009.s001.pdf]

Fig. S1

The full list of differentially expressed genes between WT and WTDk4TG skin at E14.5

FoldChange (WT/WTDk4TG)	Gene Symbol	Full Name
5.804841238	Matn3	matrilin 3
0.661896599	Pcdh10	protocadherin 10
0.551806338	Chrdl2	chordin-like 2
0.532328298	Plce1	phospholipase C, epsilon 1
0.367433623	Scye1	small inducible cytokine subfamily E, member 1
0.212172777	Hpgd	hydroxyprostaglandin dehydrogenase 15 (NAD)
0.142535014	Ibsp	integrin binding sialoprotein
0.05899705	Dkk4	dickkopf homolog 4 (Xenopus laevis)

The full list of differentially expressed genes between WT and WTDk4TG skin at E16.5

FoldChange (WT/WTDk4TG)	GeneSymbol	Full Name
15.45356205	Afp	alpha fetoprotein
9.033423668	Kng1	kininogen 1
7.233273056	EG666435	predicted gene, EG666435
4.776689754	Tssk5	testis-specific serine kinase 5
4.329379167	Fetub	fetuin beta
3.216157978	Ahsg	alpha-2-HS-glycoprotein
3.170979198	Gpx3	glutathione peroxidase 3
2.503818323	Vsig8	V-set and immunoglobulin domain containing 8
2.406854722	Ltf	lactotransferrin
2.224892093	5430431A17Rik	RIKEN cDNA 5430431A17 gene
2.217442402	Serpina1c	serine (or cysteine) peptidase inhibitor, clade A, member 1c
1.926262665	C030037D09Rik	RIKEN cDNA C030037D09 gene
1.902804734	Krt6b	keratin 6B
1.781991197	Prss16	protease, serine, 16 (thymus)
1.727831917	Sctr	secretin receptor
1.633666601	Clec1b	C-type lectin domain family 1, member b
0.662400805	Punc	putative neuronal cell adhesion molecule
0.658930819	AI197445	expressed sequence AI197445
0.633589092	Zfp711	zinc finger protein 711
0.621264646	Lrg1	leucine-rich alpha-2-glycoprotein 1
0.615305099	B930001P03Rik	RIKEN cDNA B930001P03 gene
0.598981731	Rgs18	regulator of G-protein signaling 18
0.586737388	5730408K05Rik	RIKEN cDNA 5730408K05 gene
0.586438034	I830077J02Rik	RIKEN cDNA I830077J02 gene
0.586084021	Tfpi2	tissue factor pathway inhibitor 2
0.571804329	9530098N22Rik	RIKEN cDNA 9530098N22 gene
0.563726457	Best3	bestrophin 3
0.559187166	C130079G13Rik	RIKEN cDNA C130079G13 gene

0.553048403	Rgs13	regulator of G-protein signaling 13
0.546719954	Sts	steroid sulfatase
0.543448726	1700112E06Rik	RIKEN cDNA 1700112E06 gene
0.529784484	6030440G07Rik	RIKEN cDNA 6030440G07 gene
0.524073307	Fat3	FAT tumor suppressor homolog 3 (Drosophila)
0.523894844	Tmprss11f	transmembrane protease, serine 11f
0.519650587	A530099J19Rik	RIKEN cDNA A530099J19 gene
0.513779568	Kcnd2	potassium voltage-gated channel, Shal-related family, member 2
0.512846813	Cyp2r1	cytochrome P450, family 2, subfamily r, polypeptide 1
0.510699147	Sln	sarcolipin
0.510287394	Ms4a2	membrane-spanning 4-domains, subfamily A, member 2
0.503785951	Abra	actin-binding Rho activating protein
0.500237613	OTTMUSG0000000085	predicted gene, OTTMUSG00000008540
0.498564135	Lmod2	leiomodin 2 (cardiac)
0.496199115	C1ql4	complement component 1, q subcomponent-like 4
0.492620544	Mstn	myostatin
0.489387629	4930500O09Rik	RIKEN cDNA 4930500O09 gene
0.483640848	Mill1	MHC I like leukocyte 1
0.460322502	EG433016	predicted gene, EG433016
0.452705823	Il22ra2	interleukin 22 receptor, alpha 2
0.452189956	Epha3	Eph receptor A3
0.44346488	Slc6a20a	solute carrier family 6 (neurotransmitter transporter), member 20A
0.43717567	EG408196	predicted gene, EG408196
0.437043835	RP23-198H7.1	skint 6
0.434263381	Cyp2b9	cytochrome P450, family 2, subfamily b, polypeptide 9
0.401422642	Gm1381	gene model 1381, (NCBI)
0.358282965	A430090E18Rik	RIKEN cDNA A430090E18 gene
0.336947659	Serpina12	serine (or cysteine) peptidase inhibitor, clade A (alpha-1 antiproteinase, antitrypsin), member 12
0.333864177	Clec12b	C-type lectin domain family 12, member B
0.320460438	LOC436332	similar to 60S ribosomal protein L7a (Surfeit locus protein 3) (PLA-X polypeptide)
0.319246068	Ear5	eosinophil-associated, ribonuclease A family, member 5
0.248436712	Kcne4	potassium voltage-gated channel, Isk-related subfamily, gene 4
0.240937149	9530026F06Rik	RIKEN cDNA 9530026F06 gene
0.240784572	LOC638323	hypothetical protein LOC638323
0.087228903	Dkk4	dickkopf homolog 4 (Xenopus laevis)

The full list of differentially expressed genes between WT and WTDk4TG skin at E18.5

FoldChange (WT/WTDk4TG)	GeneSymbol	Full Name
20.11667673	Krt26	keratin 26
8.473858148	Mup1	major urinary protein 1
8.407600471	A030004J04Rik	RIKEN cDNA A030004J04 gene
5.414771497	2310046K23Rik	RIKEN cDNA 2310046K23 gene

3.67606514	Krt31	keratin 31
3.562014676	Padi3	peptidyl arginine deiminase, type III
3.504836675	S100a3	S100 calcium binding protein A3
3.234048058	Ly6g6d	lymphocyte antigen 6 complex, locus G6D
3.088135384	Sprr1a	small proline-rich protein 1A
3.042195248	Acsm3	acyl-CoA synthetase medium-chain family member 3
3.03609922	Krt71	keratin 71
2.905794154	Ggt1	gamma-glutamyltransferase 1
2.872160152	Krt75	keratin 75
2.731717977	Bmp8a	bone morphogenetic protein 8a
2.714219798	Krt27	keratin 27
2.714219798	Krt25	keratin 25
2.457364722	Tchh	trichohyalin
2.39469336	Krt33a	keratin 33A
2.247999281	Krt2-ps1	keratin complex 2, basic, pseudogene 1
2.021835827	Areg	amphiregulin
2.021631457	Elovl3	elongation of very long chain fatty acids (FEN1/Elo2, SUR4/Elo3, yeast)-like 3
2.01901916	Crhr1	corticotropin releasing hormone receptor 1
2.013530928	Dlx2	distal-less homeobox 2
1.986807598	Hes2	hairy and enhancer of split 2 (Drosophila)
1.869997756	Foxe1	forkhead box E1 (thyroid transcription factor 2)
1.778283601	Sct	secretin
1.68676731	Mlana	melan-A
1.628531879	Foxq1	forkhead box Q1
1.54686219	Crym	crystallin, mu
1.538864011	Myh14	myosin, heavy polypeptide 14
1.502855425	Tubb3	tubulin, beta 3
0.65781251	9330182L06Rik	RIKEN cDNA 9330182L06 gene
0.641181826	Ch25h	cholesterol 25-hydroxylase
0.626338799	Rasgrf1	RAS protein-specific guanine nucleotide-releasing factor 1
0.62021658	BC062109	cDNA sequence BC062109
0.584887672	Tnf	tumor necrosis factor
0.577787536	Crabp1	cellular retinoic acid binding protein I
0.576734529	Lce3f	late cornified envelope 3F
0.5518977	Mpped1	metallophosphoesterase domain containing 1
0.469816631	EG434249	predicted gene, EG434249
0.425598924	Ibsp	integrin binding sialoprotein
0.392794576	Mrgprb1	MAS-related GPR, member B1
0.366460105	9530008L14Rik	RIKEN cDNA 9530008L14 gene
0.357889312	Gp1ba	glycoprotein 1b, alpha polypeptide
0.096716101	Dkk4	dickkopf homolog 4 (Xenopus laevis)

The full list of differentially expressed genes between WT and WTDk4TG skin at p1

FoldChange (WT/WTDk4TG)	GeneSymbol	Full Name
47.77830865	Krtap15	keratin associated protein 15
40.91653028	Krtap16-3	keratin associated protein 16-3
37.52345216	Krtap14	keratin associated protein 14
35.85514521	AY026312	cDNA sequence AY026312
24.94387628	Krtap6-1	keratin associated protein 6-1
19.43634597	Krtap6-2	keratin associated protein 6-2
17.09693965	Krtap16-7	keratin associated protein 16-7
16.94915254	Krtap16-5	keratin associated protein 16-5
14.6627566	1110025L11Rik	RIKEN cDNA 1110025L11 gene
9.071117562	1110032D16Rik	RIKEN cDNA 1110032D16 gene
8.422471153	4733401N17Rik	RIKEN cDNA 4733401N17 gene
7.525020694	Tchhl1	trichohyalin-like 1
4.831151263	Krtap3-2	keratin associated protein 3-2
4.774409167	Krtap9-1	keratin associated protein 9-1
4.761904762	OTTMUSG0000000049	predicted gene, OTTMUSG00000004966
4.703226413	Krtap4-7	keratin associated protein 4-7
4.506128335	4733401I12Rik	RIKEN cDNA 4733401I12 gene
4.467676362	4732454E20Rik	RIKEN cDNA 4732454E20 gene
4.387311894	Krt86	keratin 86
4.211412929	Actbl2	actin, beta-like 2
4.188306249	2310043L02Rik	RIKEN cDNA 2310043L02 gene
4.170141785	Gykl1	glycerol kinase-like 1
4.083966348	LOC665891	PREDICTED: Mus musculus hypothetical protein LOC665891 (LOC665891), mRNA (possibly wrong strand)
4.040240798	4930515B02Rik	RIKEN cDNA 4930515B02 gene
4.032745897	Krtap5-1	keratin associated protein 5-1
4.031607805	Atp12a	ATPase, H+/K+ transporting, nongastric, alpha polypeptide
4.017354973	Krtap5-4	keratin associated protein 5-4
3.94368419	2300006N05Rik	RIKEN cDNA 2300006N05 gene
3.927575508	Pdia2	protein disulfide isomerase associated 2
3.855198735	Krtap5-3	keratin associated protein 5-3
3.786874692	LOC675238	similar to hCG2043579
3.607894072	LOC672449	similar to RNA exonuclease 4 (Exonuclease XPMC2) (Prevents mitotic catastrophe 2 protein homolog)
3.488209851	LOC386392	similar to keratin associated protein 11-1
3.275466754	LOC100045026	butyrophilin-like
3.222480021	Lyg2	lysozyme G-like 2
3.195296524	Pla2g2e	phospholipase A2, group IIE
3.129204869	LOC669576	hypothetical protein LOC669576
3.018867925	Kcne1	potassium voltage-gated channel, Isk-related subfamily, member 1
2.987750224	Gprc5d	G protein-coupled receptor, family C, group 5, member D
2.924318634	OTTMUSG0000000021	predicted gene, OTTMUSG00000002196

2.860575548	Otop2	otopetrin 2
2.80788454	A030005K14Rik	RIKEN cDNA A030005K14 gene
2.752546105	Krtap13-1	keratin associated protein 13-1
2.743559494	Spred3	sprouty-related, EVH1 domain containing 3
2.711937951	5530401N06Rik	RIKEN cDNA 5530401N06 gene
2.68708854	Krt34	keratin 34
2.626119383	Crym	crystallin, mu
2.547251516	A030005L19Rik	RIKEN cDNA A030005L19 gene
2.521241459	2310040M23Rik	RIKEN cDNA 2310040M23 gene
2.492522433	Krtap5-2	keratin associated protein 5-2
2.449899554	Krtap2-4	keratin associated protein 2-4
2.334703026	Pcdhgb4	protocadherin gamma subfamily B, 4
2.320831786	Ly6g6d	lymphocyte antigen 6 complex, locus G6D
2.290111299	Cyp3a25	cytochrome P450, family 3, subfamily a, polypeptide 25
2.25840692	Vsig8	V-set and immunoglobulin domain containing 8
2.239842315	Capn8	calpain 8
2.236636099	Ramp3	receptor (calcitonin) activity modifying protein 3
2.23284062	A030004J04Rik	RIKEN cDNA A030004J04 gene
2.18603126	Krt82	keratin 82
2.172732211	Muc15	mucin 15
2.170468604	Krt2-ps1	keratin complex 2, basic, pseudogene 1
2.165674066	Gpr143	G protein-coupled receptor 143
2.158102596	Mkiaa4035	
2.143438933	Krt31	keratin 31
2.136934781	C8g	complement component 8, gamma subunit
2.124766276	Krtap17-1	keratin associated protein 17-1
2.116043844	Krt33a	keratin 33A
2.099869808	Gm312	gene model 312, (NCBI)
2.078785989	Tnfrsf13c	tumor necrosis factor receptor superfamily, member 13c
2.065176986	Serpinb13	serine (or cysteine) peptidase inhibitor, clade B (ovalbumin), member 13
2.021263694	2510049J12Rik	RIKEN cDNA 2510049J12 gene
2.016535592	Fxyd4	FXYD domain-containing ion transport regulator 4
2.01503214	2810037O22Rik	RIKEN cDNA 2810037O22 gene
2.003245257	Cryba4	crystallin, beta A4
1.986452395	Krt33b	keratin 33B
1.974762535	1700034K08Rik	RIKEN cDNA 1700034K08 gene
1.965988401	S100a3	S100 calcium binding protein A3
1.958940605	Tubb3	tubulin, beta 3
1.956219801	Sprr1a	small proline-rich protein 1A
1.885156279	Fgf5	fibroblast growth factor 5
1.821327748	Tchh	trichohyalin
1.811856791	Padi1	peptidyl arginine deiminase, type I

1.807272464	4732473B16Rik	RIKEN cDNA 4732473B16 gene
1.805673426	Sprr4	small proline-rich protein 4
1.788588803	2310046K23Rik	RIKEN cDNA 2310046K23 gene
1.783421315	Slain1	SLAIN motif family, member 1
1.772201251	Gpx2	glutathione peroxidase 2
1.769473051	Krt6a	keratin 6A
1.740947075	Dusp2	dual specificity phosphatase 2
1.71718039	Krt27	keratin 27
1.700477834	LOC16697	keratin associated protein LOC16697
1.698773486	Cldn3	claudin 3
1.6616264	Capn12	calpain 12
1.622849724	Diras2	DIRAS family, GTP-binding RAS-like 2
1.617573317	D730001G18Rik	RIKEN cDNA D730001G18 gene
1.61108426	Atp1b1	ATPase, Na+/K+ transporting, beta 1 polypeptide
1.598823266	Krt71	keratin 71
1.591545709	Krt75	keratin 75
1.57768522	Sp6	trans-acting transcription factor 6
1.576367104	St14	suppression of tumorigenicity 14 (colon carcinoma)
1.567766716	Krt36	keratin 36
1.542828931	Zbtb7a	zinc finger and BTB domain containing 7a
1.541235763	Trpm1	transient receptor potential cation channel, subfamily M, member 1
1.540808308	Ctps	cytidine 5'-triphosphate synthase
1.533718808	Wnt11	wingless-related MMTV integration site 11
1.507090863	Krt28	keratin 28
0.581179096	Tm6sf2	transmembrane 6 superfamily member 2
0.573628025	9330182L06Rik	RIKEN cDNA 9330182L06 gene
0.515299234	Nptx1	neuronal pentraxin 1
0.511503719	Hoxd1	homeo box D1
0.053195083	Dkk4	dickkopf homolog 4 (Xenopus laevis)
