# Supplementary material for: Dkk4 and Eda Regulate Distinctive Developmental Mechanisms for Subtypes of Mouse Hair
Source: PLoS One. 2010 Apr 1;5(4):e10009. doi: 10.1371/journal.pone.0010009 (PMC2850388; doi:10.1371/journal.pone.0010009)
Supplement: Figure S2 — The full list of differentially expressed genes between Ta and TaDk4TG skin (0.05 MB PDF) [file pone.0010009.s002.pdf]

Fig S2

The full list of differentially expressed genes between Ta and TaDk4TG skin at E16.5

FoldChange (Ta/TaDk4TG)	Gene Symbol	Full Name
27.56013	Shh	sonic hedgehog
4.6255	Dkk1	dickkopf homolog 1 (Xenopus laevis)
3.84644	Lgr6	leucine-rich repeat-containing G protein-coupled receptor 6
2.96841	Gli1	GLI-Kruppel family member GLI1
2.94604	Tmem16e	transmembrane protein 16E
2.93823	Ptch2	patched homolog 2
2.37934	Ptch1	patched homolog 1
2.28	Lef1	lymphoid enhancer binding factor 1
1.73363	Scube1	signal peptide, CUB domain, EGF-like 1
1.71949	Cxcr4	chemokine (C-X-C motif) receptor 4
1.67796	Tcf7	transcription factor 7, T-cell specific
1.58511	Rgs2	regulator of G-protein signaling 2
1.55928	Id3	inhibitor of DNA binding 3
1.55348	Gprasp2	G protein-coupled receptor associated sorting protein 2
1.52913	ND6	NADH dehydrogenase subunit 6
1.52651	3110082D06Rik	RIKEN cDNA 3110082D06 gene
1.51829	Rhpn2	rhophilin, Rho GTPase binding protein 2
1.50677	OTTMUSG00000003947	predicted gene, OTTMUSG00000003947
0.63035	Sphkap	SPHK1 interactor, AKAP domain containing
0.59259	E030049G20Rik	RIKEN cDNA E030049G20 gene
0.5918	Agrp	agouti related protein
0.57492	Col8a1	collagen, type VIII, alpha 1
0.56103	6430704M03Rik	RIKEN cDNA 6430704M03 gene
0.48527	Itgbl1	integrin, beta-like 1
0.05291	Dkk4	dickkopf homolog 4 (Xenopus laevis)

The full list of differentially expressed genes between Ta and TaDk4TG skin at E17.5

FoldChange (Ta/TaDk4TG)	Gene Symbol	Full Name
59.79099	Shh	sonic hedgehog
16.6657	A030013N09Rik	RIKEN cDNA A030013N09 gene
11.01398	1100001G20Rik	RIKEN cDNA 1100001G20 gene
10.78177	AW121567	expressed sequence AW121567
5.34283	Dkk1	dickkopf homolog 1 (Xenopus laevis)
5.22339	Fgf20	fibroblast growth factor 20

5.17881	Hes5	hairy and enhancer of split 5 (Drosophila)
5.081	Galnt9	UDP-N-acetyl-alpha-D-galactosamine:polypeptide N-acetylgalactosaminyltransferase 9
4.95162	Ptch1	patched homolog 1
4.46346	Padi1	peptidyl arginine deiminase, type I
4.44522	Slco4a1	solute carrier organic anion transporter family, member 4a1
4.41451	Ptch2	patched homolog 2
4.32188	Mup2	major urinary protein 2
4.16365	Edar	ectodysplasin-A receptor
3.98576	Gli1	GLI-Kruppel family member GLI1
3.8796	Clec12b	C-type lectin domain family 12, member B
3.84563	Lhx2	LIM homeobox protein 2
3.70205	Trps1	trichorhinophalangeal syndrome I (human)
3.32548	Flg	filaggrin
3.26728	Serpinb3c	serine (or cysteine) peptidase inhibitor, clade B, member 3C
3.06105	Ndph	Norrie disease homolog
3.01533	Flg2	filaggrin family member 2
2.89009	Spink3	serine peptidase inhibitor, Kazal type 3
2.54031	Krt79	keratin 79
2.48165	Cnnm1	cyclin M1
2.42795	Tcf7	transcription factor 7, T-cell specific
2.4038	Lef1	lymphoid enhancer binding factor 1
2.31573	2610307P16Rik	RIKEN cDNA 2610307P16 gene
2.31315	Slc45a2	solute carrier family 45, member 2
2.31203	Cxcr4	chemokine (C-X-C motif) receptor 4
2.3092	Alox12e	arachidonate lipoxygenase, epidermal
2.30675	A330094K24Rik	RIKEN cDNA A330094K24 gene
2.29725	Nsg2	neuron specific gene family member 2
2.27821	Etv4	ets variant gene 4 (E1A enhancer binding protein, E1AF)
2.19262	Sostdc1	sclerostin domain containing 1
2.18861	Srcs3	v-src suppressed transcript 3
2.14246	A430010J10Rik	RIKEN cDNA A430010J10 gene
2.12471	Rhpn2	rhophilin, Rho GTPase binding protein 2
2.06175	Has3	hyaluronan synthase 3
2.00515	Zfp64	zinc finger protein 64
1.99457	Plau	plasminogen activator, urokinase
1.9548	Hhip	Hedgehog-interacting protein
1.95286	I830077J02Rik	RIKEN cDNA I830077J02 gene

1.90941	Kcnh3	potassium voltage-gated channel, subfamily H (eag-related), member 3
1.8817	4933417O08Rik	RIKEN cDNA 4933417O08 gene
1.8687	Nkd1	naked cuticle 1 homolog (Drosophila)
1.86165	Rtp4	receptor transporter protein 4
1.84241	Slc22a4	solute carrier family 22 (organic cation transporter), member 4
1.82414	Gem	GTP binding protein (gene overexpressed in skeletal muscle)
1.81865	Col23a1	collagen, type XXIII, alpha 1
1.80932	Grasp	GRP1 (general receptor for phosphoinositides 1)-associated scaffold protein
1.79447	Lypd1	Ly6/Plaur domain containing 1
1.79026	Mlana	melan-A
1.77244	A730089K16Rik	RIKEN cDNA A730089K16 gene
1.76397	Ovol2	ovo-like 2 (Drosophila)
1.76164	Cdh3	cadherin 3
1.74863	Chst2	carbohydrate sulfotransferase 2
1.74705	Amdhd1	amidohydrolase domain containing 1
1.74002	2310015K22Rik	RIKEN cDNA 2310015K22 gene
1.71647	Mmp3	matrix metallopeptidase 3
1.71238	D630040I23Rik	RIKEN cDNA D630040I23 gene
1.71204	2010300C02Rik	RIKEN cDNA 2010300C02 gene
1.70802	Loxl4	lysyl oxidase-like 4
1.70405	2900022M12Rik	RIKEN cDNA 2900022M12 gene
1.69401	Rac3	RAS-related C3 botulinum substrate 3
1.69388	Sox4	SRY-box containing gene 4
1.69205	Slc24a5	solute carrier family 24, member 5
1.69154	Ell3	elongation factor RNA polymerase II-like 3
1.67769	Gabrp	gamma-aminobutyric acid (GABA-A) receptor, pi
1.66987	Egfl6	EGF-like-domain, multiple 6
1.66809	A430107O13Rik	RIKEN cDNA A430107O13 gene
1.66639	Hist3h2ba	histone cluster 3, H2ba
1.64445	Tpbg	trophoblast glycoprotein
1.64164	3110043A19Rik	RIKEN cDNA 3110043A19 gene
1.63627	EG236749	predicted gene, EG236749
1.62904	Lrp4	low density lipoprotein receptor-related protein 4
1.6271	Efnb3	ephrin B3
1.62576	Glis1	GLIS family zinc finger 1
1.6245	Slitrk6	SLIT and NTRK-like family, member 6
1.62433	Lamc2	laminin, gamma 2

1.62325	2010010I01Rik	RIKEN cDNA 2010010I01 gene
1.62319	Cbr2	carbonyl reductase 2
1.62138	S100a4	S100 calcium binding protein A4
1.62138	Pitx2	paired-like homeodomain transcription factor 2
1.60289	Kcnj4	potassium inwardly-rectifying channel, subfamily J, member 4
1.59697	Pgbd5	piggyBac transposable element derived 5
1.59168	Rasl11b	RAS-like, family 11, member B
1.58993	6330514A18Rik	RIKEN cDNA 6330514A18 gene
1.58678	Hs3st6	heparan sulfate (glucosamine) 3-O-sulfotransferase 6
1.58566	LOC230628	similar to C05G5.5
1.57204	Rgs2	regulator of G-protein signaling 2
1.5585	Wif1	Wnt inhibitory factor 1
1.55522	Sh3rf2	SH3 domain containing ring finger 2
1.53956	Lama5	laminin, alpha 5
1.53213	Cpne8	copine VIII
1.52077	Sox21	SRY-box containing gene 21
1.51411	Cacna1g	calcium channel, voltage-dependent, T type, alpha 1G subunit
1.51177	Ovol1	OVO homolog-like 1 (Drosophila)
1.51176	Cpsf4l	cleavage and polyadenylation specific factor 4-like
1.50936	Abr	active BCR-related gene
1.50716	Esr1	estrogen receptor 1 (alpha)
1.50445	Smox	spermine oxidase
1.50064	Mycl1	v-myc myelocytomatosis viral oncogene homolog 1, lung carcinoma derived (avian)
1.50027	Iqcd	IQ motif containing D
0.66062	Cthrc1	collagen triple helix repeat containing 1
0.65447	Snca	synuclein, alpha
0.64137	Sgk1	serum/glucocorticoid regulated kinase 1
0.63009	Larp2	La ribonucleoprotein domain family, member 2
0.62868	Gsta4	glutathione S-transferase, alpha 4
0.62443	Slc24a3	solute carrier family 24 (sodium/potassium/calcium exchanger), member 3
0.61669	Anxa3	annexin A3
0.6023	Sned1	sushi, nidogen and EGF-like domains 1
0.59123	Cfh	complement component factor h
0.59087	Csrp2	cysteine and glycine-rich protein 2
0.58258	Rasd1	RAS, dexamethasone-induced 1
0.58174	Gpr111	G protein-coupled receptor 111
0.57106	Slc4a8	solute carrier family 4 (anion exchanger), member 8

0.54534	EG244911	predicted gene, EG244911
0.53392	Tgfa	transforming growth factor alpha
0.53037	Ramp3	receptor (calcitonin) activity modifying protein 3
0.50632	Srd5a2l2	steroid 5 alpha-reductase 2-like 2
0.50143	Grid2	glutamate receptor, ionotropic, delta 2
0.48313	Car2	carbonic anhydrase 2
0.4605	Mx1	myxovirus (influenza virus) resistance 1
0.4605	BC038167	cDNA sequence BC038167
0.43757	C430042M11Rik	RIKEN cDNA C430042M11 gene
0.43477	Slc39a8	solute carrier family 39 (metal ion transporter), member 8
0.41628	Cadps	Ca2+-dependent secretion activator
0.38408	1110017D15Rik	RIKEN cDNA 1110017D15 gene
0.3789	Tubb3	tubulin, beta 3
0.37594	Scgn	secretagogin, EF-hand calcium binding protein
0.36671	Apol7a	apolipoprotein L 7a
0.35727	AA467197	expressed sequence AA467197
0.3321	Sgk2	serum/glucocorticoid regulated kinase 2
0.32948	Atp1b1	ATPase, Na+/K+ transporting, beta 1 polypeptide
0.28452	Krt19	keratin 19
0.08519	Gpx3	glutathione peroxidase 3
0.0789	3110079O15Rik	RIKEN cDNA 3110079O15 gene
0.07081	C130073F10Rik	RIKEN cDNA C130073F10 gene
0.0645	Lce3f	late cornified envelope 3F
0.06245	2300002M23Rik	RIKEN cDNA 2300002M23 gene
0.05194	Dkk4	dickkopf homolog 4 (Xenopus laevis)
0.02967	Saa1	serum amyloid A 1
0.02438	Col2a1	collagen, type II, alpha 1
0.0168	Fetub	fetuin beta
0.00245	2310057N15Rik	RIKEN cDNA 2310057N15 gene
0.00212	EG546672	predicted gene, EG546672
0.00193	2310034C09Rik	RIKEN cDNA 2310034C09 gene
0.00185	4930553J12Rik	RIKEN cDNA 4930553J12 gene
0.00154	EG433047	predicted gene, EG433047
0.00112	Krtap13	keratin associated protein 13
