# Supplementary material for: Dkk4 and Eda Regulate Distinctive Developmental Mechanisms for Subtypes of Mouse Hair
Source: PLoS One. 2010 Apr 1;5(4):e10009. doi: 10.1371/journal.pone.0010009 (PMC2850388; doi:10.1371/journal.pone.0010009)
Supplement: Figure S3 — Expression levels of Sox2, Sox18, CD133, Noggin and Troy in Ta and TaDk4TG skin at E16.5. (0.03 MB PDF) [file pone.0010009.s003.pdf]

Fig. S3

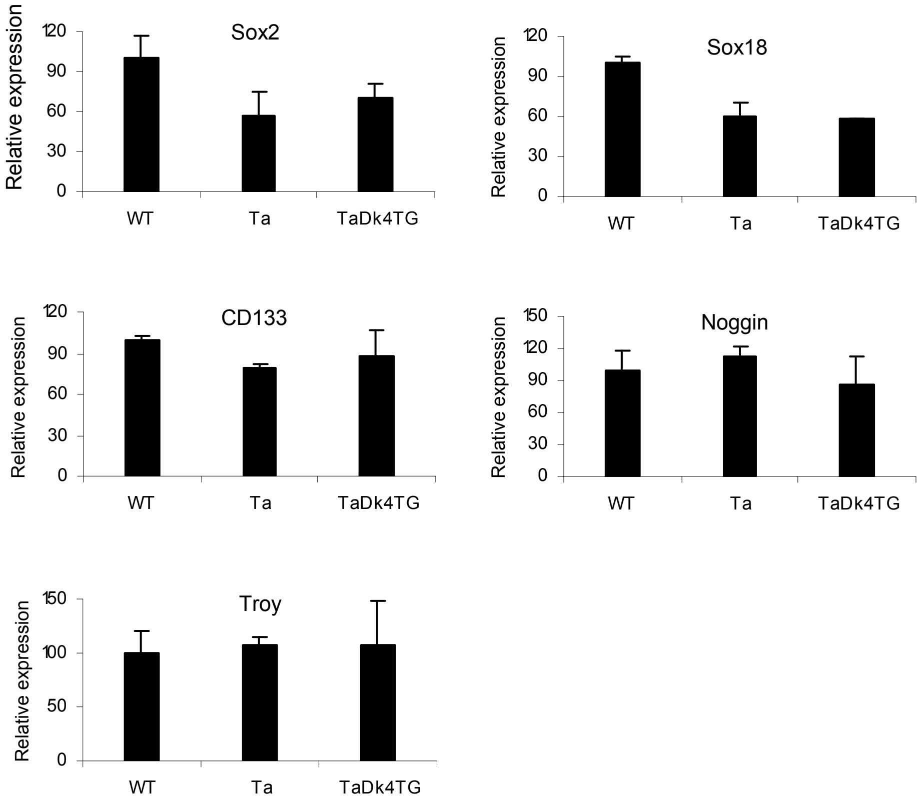

Fig. S3: Expression levels of Sox2, Sox18, CD133, Noggin and Troy in Ta and TaDk4TG skin at E16.5. Sox2 and Sox18 were significantly down-regulated in Ta skin, but no further expression changes were seen in TaDk4TG skin. CD133, Noggin and Troy were normally expressed in Ta or TaDk4TG skin.
