# Supplementary material for: Dkk4 and Eda Regulate Distinctive Developmental Mechanisms for Subtypes of Mouse Hair
Source: PLoS One. 2010 Apr 1;5(4):e10009. doi: 10.1371/journal.pone.0010009 (PMC2850388; doi:10.1371/journal.pone.0010009)
Supplement: Table S1 — Expression levels of Wnt pathway genes in Ta and TaDk4TG skin at E16.5 (0.04 MB DOC) [file pone.0010009.s004.doc]

**Table. S1**

|  | **Relative expression levels** | |
| --- | --- | --- |
| **Genes** | **Ta** | **TaDk4TG** |
| *Wnt3* | 8.7±1 | 10.0±1.3 |
| *Wnt3a* | 9.5±1.4 | 15.1±3.4 |
| *Wnt4* | 7.1±0.3 | 10.3±1.9 |
| *Wnt5a* | 5.3±0.02 | 6.0±0.3 |
| *Wnt6* | 6.7±0.3 | 7.3±2.3 |
| *Wnt7a* | 4.2±0.6 | 3.8±0.5 |
| *Wnt7b* | 22.6±4.1 | 20.7±4.1 |
| *Wnt10a* | 4.1±0.6 | 5.7±0.5 |
| *Wnt10b* | 6.5±0.5 | 6.1±0.4 |
| *Wnt11* | 5.8±1.4 | 4.9±0.7 |
| *Fzd1* | 6.6±0.6 | 6.4±0.9 |
| *Fzd2* | 15.6±0.3 | 11.8±1.1 |
| *Fzd3* | 7.1±0.3 | 6.0±1.5 |
| *Fzd4* | 4.8±1.5 | 4.5±1.2 |
| *Fzd5* | 3.5±1.2 | 3.1±0.1 |
| *Fzd6* | 10.8±1.1 | 11.8±1.7 |
| *Fzd7* | 3.8±0.8 | 2.9±0.1 |
| *Fzd8* | 7.6±1.2 | 6.7±0.8 |
| *Fzd9* | 11.3±1.1 | 9.2±0.04 |
| *Fzd10* | 9.2±0.5 | 7.5±0.7 |
| *Lrp5* | 6.9±0.6 | 6.0±0.1 |
| *Lrp6* | 6.2±0.1 | 5.2±0.3 |
| *Kremen1* | 10.1±0.2 | 8.9±0.8 |
| *Kremen2* | 6.4±0.4 | 4.9±0.1 |

Expression levels of Wnt pathway genes in Ta and TaDk4TG skin

at E16.5*

*: Each of the two sets of RNAs for each genotype was assayed in triplicate

by Taqman Q-PCR. Reactions were normalized to GAPDH.
